# Exploring a Fuzzy Rule Inferred ConvLSTM for Discovering and Adjusting the Optimal Posture of Patients with a Smart Medical Bed

**DOI:** 10.3390/ijerph18126341

**Published:** 2021-06-11

**Authors:** Francis Joseph Costello, Min Gyeong Kim, Cheong Kim, Kun Chang Lee

**Affiliations:** 1 SKK Business School, Sungkyunkwan University, Seoul 03063, Korea; f.costello@g.skku.edu (F.J.C.); webser2@g.skku.edu (M.G.K.); saga@g.skku.edu (C.K.); 2 Predictive Analytics and Data Science, Economics Department, Airports Council International (ACI) World, Montreal, QC H4Z 1G8, Canada; 3 Samsung Advanced Institute for Health Sciences & Technology (SAIHST), Sungkyunkwan University, Seoul 03063, Korea

**Keywords:** smart medical bed, health information technology, ConvLSTM, fuzzy inference, clinical healthcare, public health

## Abstract

Several countries nowadays are facing a tough social challenge caused by the aging population. This public health issue continues to impose strain on clinical healthcare, such as the need to prevent terminal patients’ pressure ulcers. Provocative approaches to resolve this issue include health information technology (HIT). In this regard, this paper explores one technological solution based on a smart medical bed (SMB). By integrating a convolutional neural network (CNN) and long-short term memory (LSTM) model, we found this model enhanced performance compared to prior solutions. Further, we provide a fuzzy inferred solution that can control our proposed proprietary automated SMB layout to optimize patients’ posture and mitigate pressure ulcers. Therefore, our proposed SMB can allow autonomous care to be given, helping prevent medical complications when lying down for a long time. Our proposed SMB also helps reduce the burden on primary caregivers in fighting against staff shortages due to public health issues such as the increasing aging population.

## 1. Introduction

This paper presents an advanced health information technology (HIT)-based smart medical bed (SMB) prototype. For over 100 years, electronic medical beds have been one of the leading clinical healthcare features and are essential for the healthcare environment [1]. Due to recent advances in technology, more innovative designs are paving the way for future SMBs that can help to alleviate some of the growing problems seen in public health and clinical healthcare [1]. This is because, as life expectancy increases, the prevalence of chronic illness will also rise. Thus, health managers need to regularly consider the quality of life of their patients, especially those who frequently need healthcare assistance [2]. SMBs have been seen in multiple forms, with various objectives being present in different implementations. i.e., measuring vital organs [3], 3D posture prediction with robot-assisted deep learning technology [4], and monitoring of respiratory rate [5].

With public health issues such as the aging population, current solutions for dealing with increased demand are needed. For instance, as elderly healthcare demand increases, healthcare professionals are having less time per patient. If this is to persist, an elderly individual could be left in the same position for a lengthy period; the blood flow may be cut off due to the friction between the skin and the bed’s surface. If oxygen is continuously cut off from the cells, cell death will eventually lead to bedsores and pressure ulcers [6]. Furthermore, preventing the risk of a patient falling off a bed is also a key area of research. Both of these issues require increased amounts of care. However, there is a growing shortage of healthcare professionals. This trend has left a gap between supply and demand, and thus clinical healthcare has started to turn towards technology-driven innovations and robotics. Such HIT is anticipated to tackle this public health issue by providing options for reform in clinical healthcare services and care provision [7,8].

A prior literature review (see Table 1) shows that one area that reveals noticeable results is pressure prediction and ulcer prevention. If successfully implemented, such techniques can go a long way in delegating roles and responsibilities from the human to the agentic smart medical bed. For example, the kNN clustering algorithm has shown promising results (i.e., [9,10,11,12]). The most prominent example saw Yousefi et al. [13] use a tiled architecture SMB, which used changes within an air bladder to adjust the bed to patients’ bodies. Overall, this showed high accuracy in predicting the patients’ posture [13].

Machine learning approaches have also been attempted. This was seen in the use of support vector machines (SVMs). SVMs have been utilized in either a discrete fashion [17] or embedded into a broader model, such as principal component analysis [19] and fuzzy logic [20]. The methodology of Ren et al. [20] methodology was the most effective example whereby a very high accuracy was observed based on 20 unique postures. Artificial neural networks (ANN) have also been explored. Based on 25 features obtained from a proprietary SMB, feature selection was utilized before data was fed to the ANN. Overall, the model was highly accurate in predicting patient intentions. Although this research was not directly on ulcer prevention, it could be implemented for such use [21]. The last approach identified in our literature search was the use of deep learning. Ulcer prevention was explored through ConvNets [4] and autoencoders [9], with the latter showing the highest accuracy when predicting four unique postures.

Other research into SMBs was also interesting, but were not included in the table, as these studies cannot be directly compared with this one. This research on SMBs saw Viriyavit and Sornlertlamvanich [22] implement a Bayesian network and neural network to predict bed position as a preventative measure against falling off the side of the bed. Used as an early warning signal, they achieved high accuracy in predicting the prevention of falling. However, as acknowledged by the authors, the data sample was a small number of sensors and samples. Thus, they concluded that the results were promising despite needing much future work [22]. Another interesting concept was seen in Davoodnia et al. [23]. They used a multitask deep learning algorithm using a specific feature selection of combined datasets to predict body mass index (BMI) in people lying on an SMB. In doing so, they wanted to show the viability of using sensors as an effective way to measure BMI. They successfully proved their concept for personalized healthcare within a smart home [23].

One noticeable omission from prior literature is the attempt to build a solution that allows for this task to be delegated away from the human caregiver. Besides warnings about potential pressure dangers or signs of potential falls from the bed, current methods do not replace the caregivers’ physical roles and responsibilities. For patients that regularly need assistance [2], the time-based management of patients is paramount. However, the methods used in the literature, shown in Table 1, till rely on human interaction to carry out the duties. These tools successfully alert caregivers to where pressure is, but do not assist in patients’ movement. A more unified approach is lacking whereby both patients and caregivers receive benefits. This is because prior research has been patient-focused, sometimes leaving the care workers’ perspectives behind.

**Figure 1:**
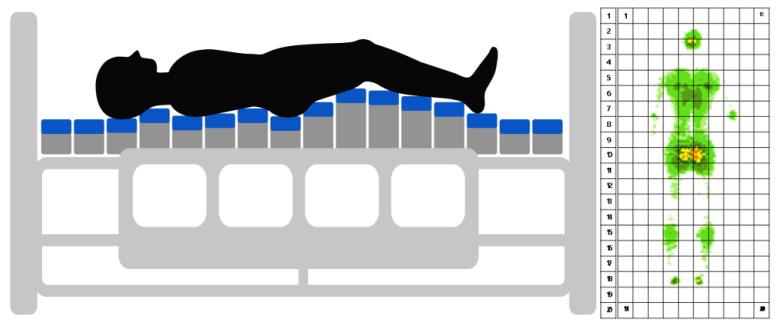
Example of a patient laying down on our proposed smart bed. As can be seen, the bed is designed to have an individual adjustable matrix layout that functions alongside imbedded sensors that continuously collect data of the patient’s posture. The sensor layout has been shown in the form of a grid matrix where each box represents a sensor (Courtesy: Ninebell Co., Ltd., Seongnam, Korea).

The proposed SMB attempts to rectify this issue and move towards an agentic system, allowing for the delegation of physical duties away from the clinical caregiver, providing a system that predicts and prevents complications such as pressure ulcers and automatically adjusting the bed’s position in a timely manner. We explore the use of a fuzzy inferred convolutional neural network (CNN) and Long short-term memory model (LSTM) along with sensors that are embedded in a horizontally partitioned bed (HoPB) (See Figure 1). Thus, this paper aims to explore the use of a ConvLSTM to predict a patient’s posture and pressure on a matrix. Next, the fuzzy inference system adjusts the beds’ dimensions to disperse any high pressure on the bed.

## 2. Methodology

### 2.1. Proposed Smart Medical Bed’s Structure and Sensor Positioning

As shown in Figure 1, the proposed SMB consists of twenty HoPBs containing ten sensors each. This formulation provides a grid-like structure that consists of an array of 10–20 sensors. Furthermore, each HoPB can move up and down by 40 mm. This movement is based on a robotic mechanism upon which each HoPB sits. Each HoPB can be moved independently of any other, so that each patient’s position can be unique. This paper employs a ConvLSTM to analyze the patient’s posture over a time series using this sensor configuration. A fuzzy inference mechanism can then automatically decrease or raise each HoPB depending on the danger of specific medical conditions (i.e., pressure ulcers). These concepts, including the obtained data and data preprocessing needed, will be presented in the following sections.

### 2.2. Dataset and Feature Preprocessing

When writing this manuscript, the proposed smart medical bed was still in its production phase, and thus data was not yet obtainable. To rectify this issue, we obtained the publicly available PmatData dataset [24]. We adapted this to fit the dimensions of the sensor placement that would be used within our proposed smart medical bed. This data was then used to train the ConvLSTM model. It was obtained from 13 subjects using a pressure mapping system embedded into a mattress. The pressure sensors had a scan rate of 2048 sensors/sec and produced a total sensor array of 32 × 64. The participants were asked to adopt varied postures for two minutes each time. A total of 20,024 data points were classified into ten postures [23] and used for learning (see Table 2). The data were randomly divided into five partitions, four for training (80%, 16019) and the remaining for test data purposes (20%, 4005).

Using this public dataset, we experimentally created the ulcer pressure level. Since accurate information about ulcer levels was not obtainable from the public dataset, it was generated with the following mechanism: convolution pooling of the 32 × 64 pressure sensors in the original data was converted to a 10 × 20 matrix (See Figure 1). Next, the matrix data was converted from the pressure sensor data of the 10 × 20 matrix into heat units. These heat units provided a representation of the pressure amount at each HoPB. Lastly, based on the pressure loaded on each HoPB, a value from 0 (no contact) to 20 (maximum in total sample) was placed for each HoPB (i.e., each row of the grid in Figure 1). At this time, the average pressure level for the entire sample, except zero, was set to 10, and the ulcer level for each HoPB was assigned based on the variance from this average.

### 2.3. Fuzzy Rule Inference with ConvLSTM (FICL)

**Figure 2:**
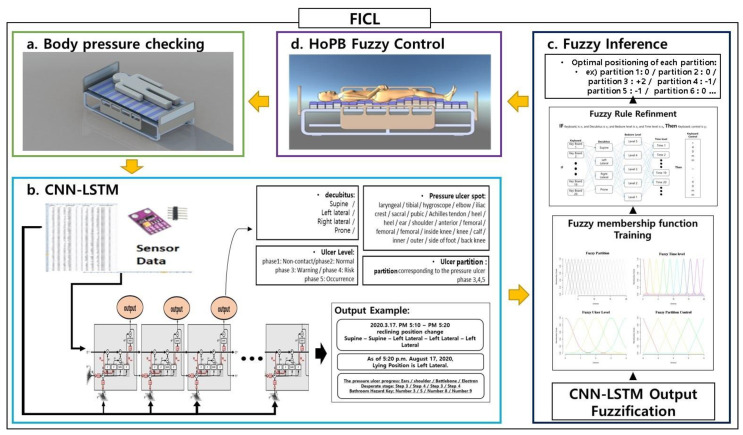
Schematic overview of the proposed FICL-based smart medical bed. This model has four stages: (**a**) body is checked with sensors embedded within the HoPB and data is collected over 80 s periods; (**b**) a multi-input ConvLSTM model then makes an analysis of the patients’ status; (**c**) this is then updated with fuzzy inference; (**d**) the HoPB of the bed is adjusted to prevent bedsores and pressure ulcers. (Ninebell Co., Ltd. provided smart bed pictures in this figure.).

For ulcer prevention (see Figure 2), we must first analyze the pressure points based on the patient’s posture. According to Lindan [25], pressure on the body should preferably be kept below 32 mmHg so that the blood circulation can flow naturally within vital arteries and nerves [25]. Continuously laying on a body part with a pressure of over 32 mmHg is when blood circulation issues arise. Although BMI has been considered as an important variable in prior work [16], the proposed mechanism in this paper deemed pressure per unit area of the HoPB more crucial. Variations in BMI do not affect the ability of these sensors to analyze pressure points on a patient’s body and adjust the HoPB accordingly. Therefore, BMI was not considered for analysis within our proposed model.

### 2.4. ConvLSTM

**Figure 3:**
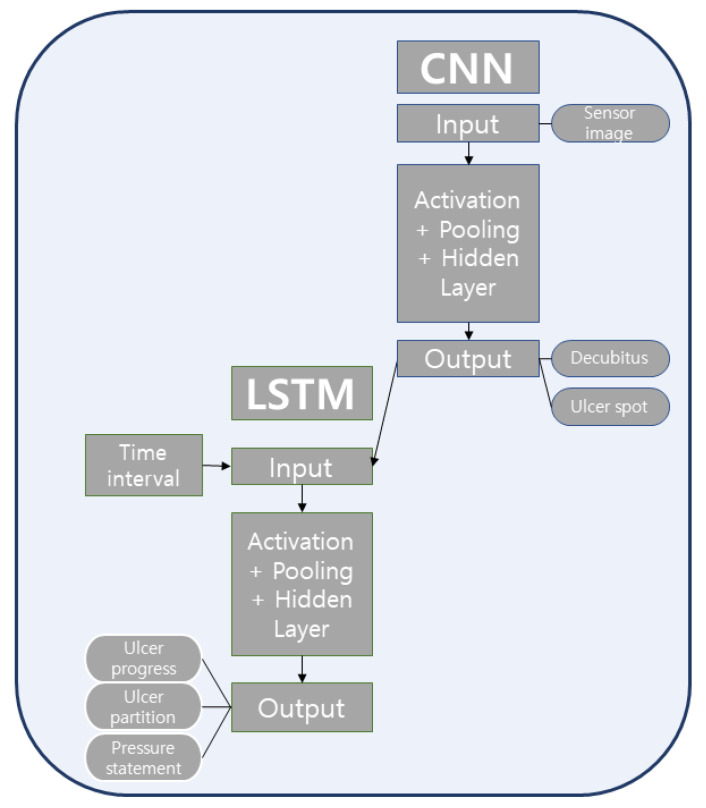
Schematic diagram of the ConvLSTM architecture used within the FICL model. This includes the multi-input data, which is separately fed into the CNN and the LSTM.

Recent work has shown the reliability of recurrent neural networks (RNNs) for analyzing data created by multiple sensors [26]. Based on this, we integrate an alternative approach for our SMB. We utilize a multi-input ConvLSTM model for prediction (see Figure 3). Once the patient lies down, data from the sensors embedded within the HoPB produces an image for the CNN, connecting to an LSTM. Next, the distribution of the body pressure is analyzed and classified into ten positions. The somatic pressure sensor’s input value is extracted by postural characteristics through the artificial neural network algorithm. This also analyzes the patient’s body shape, such as height and weight, through the patient’s body pressure. Thus, the CNN’s role can be summarized as follows:

- Recognition of posture while lying on the SMB
- Prediction of body flexure by posture position and body shape

The next section of the model is analyzed with the LSTM model. The LSTM’s role is in determining the level of pressure ulcer risk over time. The LSTM recognizes each posture position’s time and prevents the pressure ulcer from occurring due to one fixed position. It also detects the progress of the pressure ulcer and determines which HoPB should move. It then predicts the appropriate pressure ulcer risk. As a result, it uses the patient’s lying time data to detect the level of pressure ulcer risk according to the posture position and helps to control the purge inference. More specifically, the LSTM does the following:

- Recognition of time lying on the SMB in a particular posture
- Predicting the position of the HoPB which is suitable for contacting the body area: (a) predict and present the HoPB that requires movement by sensing patients’ body pressure and posture; (b) predict and present body pressure data for each HoPB used in fuzzy reasoning to adjust the position of the HoPB according to patients’ postures and body shape
- Predicting potential ulcer incidence: infer the occurrence area of the ulcer according to the lying position (suggest specific ulcer occurrence areas, for example, the shoulder blade or elbow)
- Diagnosis of ulcer progression stage: time series data analysis from the LSTM predicts ulcer progression stage by analyzing contact areas of patients’ bodies; (a) ConvLSTM algorithm learns body pressure sensor data in a time series; (b) the implemented algorithm recognizes the patients’ body pressure over time to diagnose the progress of the potential ulcer risk
- Prediction of increased ulcer risk by the body contact with the HoPB; (a) if the ulcer is in stage three or higher, immediate HoPB repositions are required; (b) the analyzed datasets are delivered to the fuzzy inference algorithm for repositioning. Next, fuzzy inference controls enable the optimized movements of the HoPB.
- Printing out the guidance: the output of the final comprehensive guide from the ConvLSTM system; (a) the upper part of the guidance provides the change in lying position; (b) the bottom part of the guidance includes the lying position status, the area where the ulcer occurred, the ulcer progress, and the HoPB in contact with the body area containing the ulcer.

## 3. Fuzzy Inference

**Figure 4:**
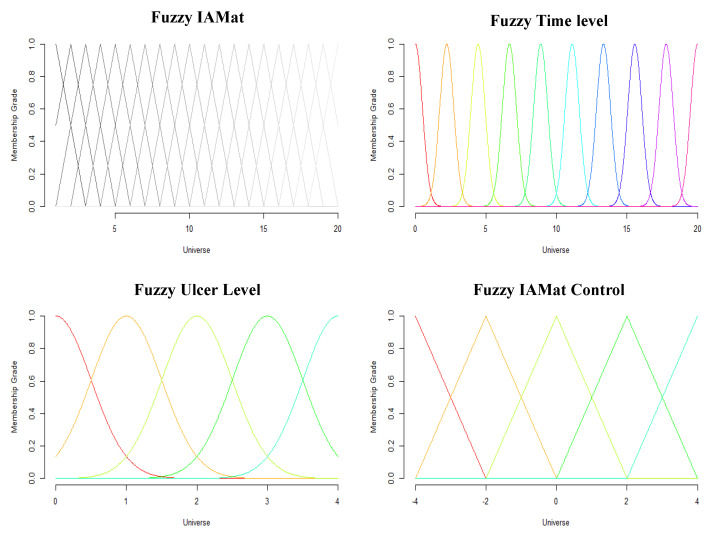
Fuzzy Membership Function of the Fuzzy Inference System used within the FICL model.

The fuzzy inference control is based on the Mamdani Fuzzy System [27,28,29] and use output data from the ConvLSTM algorithm to present an SMB optimized environment for users. The fuzzy inference algorithm performs (1) fuzzification, (2) fuzzy membership function training, (3) fuzzy rule refining, and (4) HoPB movement optimization function. Researchers initially produce fuzzy membership (see Figure 4) functions based on a defined universe. The universe acts as the *x*-axis where fuzzy set memberships can take place within the *y*-axis. Within the universe, fuzzy sets and membership functions are optimized through a series of processes presented in the Adaptive-network-based Fuzzy Reference System (ANFIS).

Further, the purging process is essential for efficient machine operation. There are about 8000 fuzzy rules needed to determine the movement of each HoPB on the bed. The bed goes through purge rules to make it work without unnecessary disturbance. The process of (2) and (3) is to optimize the HoPB movement.

The fuzzy inference process is as follows (see Figure 3 in the Fuzzy inference segment of Figure 2). Step 1 is the input fuzzification. Step 2 matches the input value to each fuzzy membership function and trains the membership function. Step 3 is the refining of the process of the fuzzy rule. In step 4, defuzzification of the aggregated output in a usable form is performed.

The Mamdani inference system is composed of IF-THEN rules in the form “IF X is A THEN Y is B”, such as “IF PRESSURE is HIGH THEN ULCER RISK is High”. The IF rule is called the antecedent, and the THEN part is called the consequence of the rule. Most Mamdani systems contain several IF-THEN rules. Typically, each of the rules might use different fuzzy sets, Ak and Bk. The antecedent parts and consequent parts can be combined with AND and OR logic operators. Although fuzzy rules can be expressed through AND rules and OR rules, this study only used AND rules to minimize overlaps that occur when using OR rules. In the Mamdani fuzzy system, AND rules are calculated as follows:

Membership[(X is C AND X is D│X = x)] 
= min[(Membership(X is c│X = x), Membership(X is D│X = x))](1)

= min[(μC(x), μD(x))](2)

Fuzzy rules used in this study are as follows:

- R_1_: *IF* HoPB is HoPB1 and Decubitus is Supine and Time level is Time1 and Ulcer Level is Phase 1 *THEN* HoPB Control is Not Moving.
- R_2_: *IF* HoPB is HoPB1 and Decubitus is Supine and Time level is Time1 and Ulcer Level is Phase 2 *THEN* HoPB Control is Not Moving.
- R_3_: *IF* HoPB is HoPB1 and Decubitus is Supine and Time level is Time1 and Ulcer Level is Phase 3 *THEN* IAM at Control is Not Moving.
- R_4_: *IF* HoPB is HoPB1 and Decubitus is Supine and Time level is Time1 and Ulcer Level is Phase 4 *THEN* HoPB Control is Slight Descent.
- R_5_: *IF* HoPB is HoPB1 and Decubitus is Supine and Time level is Time1 and Ulcer Level is Phase 5 *THEN* HoPB Control is Slight Descent.

This fuzzy rule varies each HoPB movement depending on the respective pressure ulcer level. In step 1 of the fuzzy inference in Figure 3, a variable’s fuzzification process evaluates how satisfied each concept is with a given input. The first step is to figure out the range of preconceptions and postscripts of the rules and how much the input value belongs to a given fuzzy membership function, or fuzzy set. In a specific fuzzy rule R_k_, if X’s degree in “IF X is A THEN Y Is B” is greater than 0, the rule is called ‘fired’. In the example above, High and Low are languages modeled as fuzzy sets and membership functions that represent each concept, and can be expressed to the extent that each input concept belongs to the fuzzy set through the membership function.

In step 2, the truncated fuzzy set corresponds to the results obtained from the antecedent’s result of each rule and aggregates all the truncated fuzzy sets. A set *µ_output k|x_* is such that:

(3) $$\mu_{output\;k|x}\left(y|x\right)=\min\left(\mu_{BK}\left(y\right),\;\mu_{AK}\left(x\right)\right)$$

(4) $$\mu_{Mandani|x}\left(y\right)=max\left[\mu_{output\;k|x}\left(y\right)\right]=max\left[min\left(\mu_{Bk}\left(y\right),\mu_{Ak}\left(x\right)\right)\right]$$

For step 3, the aggregated fuzzy set must be defuzzified. In the defuzzification process, defuzzification transforms the aggregated fuzzy set and fuzzy membership function (*µ_Mamdan_i*) into one single crisp number. The Mamdani Fuzzy Inference system uses the Centre of Gravity (COG) defuzzification method. COG refers to the fuzzy set area to return the center of gravity of the fuzzy set *µ_Mamdani_*, so that it is finally collected and bound. This then returns the projection of the membership area represented by the set.

## 4. Results and Analysis

**Figure 5:**
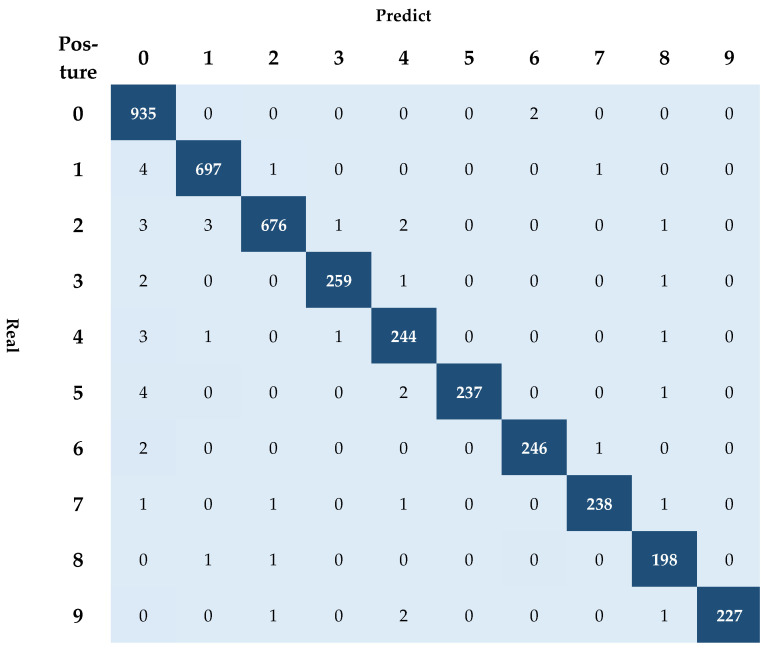
Confusion matrix of the results obtained from the ConvLSTM model.

Results obtained from this research show promising signs of the viability of using the proposed model. Although there are ten classes, the confusion matrix shows how effective the CNN-LSTM was in predicting a person’s posture and flexure. Our results showed a 98.81% weighted accuracy (see Figure 5). Table 3 provides further evidence of the power of this method by showing a weighted precision score of 98.81%, a weighted recall of 98.80%, and, lastly, a weighted F1 score of 98.80%. Although accuracy has been used as one of the primary metrics for analyzing posture, the F1 measure is also vital in providing knowledge about the model’s weakness. As our F1 measure is also very high, it gives us extra confidence in the model’s ability to decipher between each position that a patient will be able to lie on when on top of the SMB. These results are comparable with previous work (see Table 1), whereby similar results were obtained, albeit for a different purpose [23]. Based on these results, we are confident in moving to our model’s next stage, where fuzzy inference is used.

To ensure that the fuzzy controls work effectively, the HoPB number, information about the pressure ulcer level, and the patient’s lying position were used as input values for the fuzzy operation. Table 4 shows the fuzzy inference results; positive numbers show a rise in the HoPB, and negative numbers indicate a fall in the HoPB.

Updated result based on fuzzy inference: as shown in Table 5, the output of the LSTM can be seen in columns 1 to 3. From this result, fuzzy logic then updated each HoPB on of the SMB to maintain either one of the modes available. In this paper, we show the ulcer prevention mode results. As shown in the optimized setting column, the fuzzy inference has updated the HoPB positioning as a preventive measure against the risk of ulcers in the patients. As this system is automatic, based on this fuzzy inference-based adjustment the HoPB would automatically adjust, not requiring any attention from the caregiver.

The process will allow the bedsores and surrounding areas to be cared for with a separate HoPB. In the case of unconscious patients, the caregiver takes less trouble with periodically changing posture and can air the affected area through the movement of the HoPB. Besides, existing products such as air mattresses limit the coexistence of critical body pressure (32 mmHg) that causes pressure ulcers. On the other hand, dispersing body pressure using the HoPB movement can actively control the critical body pressure to prevent the patient from suffering from a pressure ulcer.

## 5. Discussion

Thanks to advances in medicine and improved environmental conditions, the extension of human life expectancy is a widely welcome recent phenomenon. However, it is also true that this has created public health challenges. According to the World Health Organization (WHO), population aging is one particular challenge, requiring an effective and sustainable strategy [30]. In particular, providing support for clinical healthcare workers in dealing with the increased demand that an aging population will create is one area of research. In this regard, this paper presents the development of a smart medical bed based on an information technology architecture. Due to its unique design and supporting software, we believe this can positively help reduce this increased demand burden. For instance, the smart medical bed proposed utilizes artificial intelligence to help predict and prevent complications while patients lie on a medical bed. Furthermore, it uses a proprietary robotic design to automatically adjust HoPB position for dispersing any pressure on a patients’ body. Such a system allows clinical caregivers to delegate these roles and responsibilities to free up their time and physical strength for other duties.

### 5.1. Experiment Results and Insights

The prediction performance (Accuracy: 98.61%, F1-score: 0.9860) of the ConvLSTM model, shown in this study, can be considered to have improved compared to previous studies (i.e., [18,19,20,24], refer to Table 1). This prediction performance has an important significance practically as it provides the SMB’s optimal posture more successfully, based on various postures. For this reason, ConvLSTM can be mounted on top of the SMB’s architecture, demonstrating a correct prediction of decubitus to a high and significant level (see Table 2). Once the posture was successfully identified from ten different postures, it was fed to the fuzzy inferred model based on twenty HoPBs. This is where fuzzy logic was successfully implemented within the architecture to help reduce pressure ulcers around a time-based system. This adjustment of all HoPBs within the SMB confirmed that the HoPB adjustment system effectively distributed the body pressure based on the patient’s natural body curve (see Table 4 and Table 5).

As shown in Table 5, the fourth HoPB is rising, balancing the cervical vertebrae. Next, the fifth and sixth HoPB are falling, reducing the shoulders and back’s upper body pressure. The seventh and eighth HoPBs also rise to relieve pressure on the spine. HoPB nine, ten, and eleven in the pelvic area are lowered in places where pressure sores occur, reducing the risk of bedsores and improving posture comfort. Next, the HoPB of the heel (No. 18) and the calf (No. 16), where bedsores are prone to occur, also used an optimized posture to prevent pressure sores in patients. By adjusting the HoPB with fuzzy logic, our study’s results provide a valuable model for preventing bedsores. It also allows for greater convenience for patients who are bed-bound for long periods.

### 5.2. Implications

The current research results have academic and practical value. Academically, this work demonstrates the robustness of ConvLSTM in classifying the correct postures based on body pressure sensor data. Prior research [23,24] using body pressure sensor data has predicted participants’ BMI and posture; however, our work has shown a more advanced result compared with these studies. Further, as we combine a CNN and LSTM, we have used the advantages of each for analyzing the SMB based on time-series data (LSTM) in the form of an image (CNN). In this way, we propose a deep learning model that can better explain more diverse and accurate factors than conventional research models (see Table 1).

Furthermore, we introduce an HoPB design so that the bed can be controlled individually depending on the patient’s position. Thus, we introduce fuzzy logic to help effectively manipulate each HoPB to benefit the patient while reducing the clinical caregivers’ workload. Our proposed method can be introduced in SMB’s that have the same HoPB functionality (currently under development), leading to technology-driven innovation in the prevention of bedsores. The combination of the ConvLSTM can also be used in SMBs that are currently being used.

To summarize, our results can be interpreted in the following ways: (a) our SMB can help clinical caregivers by providing a technological tool that delegates roles and responsibilities to prevent medical complications while patients lie on the medical bed; (b) can allow clinical caregivers to focus on providing quality care to patients that are conscious as they will not need to worry about unconscious patients’ posture, and thus medical complications, while lying; (c) allows managers to more effectively utilize staff as it removes the time and physical burden needed for these tasks.

### 5.3. Limitations and Future Work

Our research has some notable limitations that must be discussed. First, there is a limited amount of public data available for simulating our model’s potential performance. Although the obtained public data used in this study were collected from 13 subjects, future studies need to secure a broader sample to consider more subjects, age, sex, and body types. Next, the fuzzy inference results are based on data that was transformed to meet the twenty HoPB structure proposed in our SMB. This means the results cannot be verified directly from the proposed SMB sensors embedded within the HoPB. Therefore, it will be necessary to obtain data from the proposed SMB once it is completed and reanalyze a new dataset using our fuzzy inference system in a future study. After the production of the SMB is complete, another study will be needed to verify this paper’s results. In addition, most bedsores can be prevented from developing into chronic diseases through initial diagnosis and response before they occur. Extensive efforts are needed to verify whether information technology based on body pressure sensor information effectively prevents and treats chronic diseases. Therefore, a longitudinal study will be necessary to understand the actual effectiveness of such a system.

## 6. Conclusions

Using an individually adjustable matrix design that allows for the movement of the bed, we successfully explored the use of a fuzzy inferred ConvLSTM model to help to predict patient posture and prevent ulcer pressures. Overall, promising results about the viability of this model are demonstrated, especially in prediction accuracy. Furthermore, the fuzzy inference system showed promising results in adjusting the individually adjustable matrix position for further pressure reduction in specific areas of the body.

## Figures and Tables

**Table 1:** Performance of fuzzy inferred convolutional neural network (CNN) with Long short-term memory model, ConvLSTM, compared to other models.

Study	Sensor Type	Method	No. of Postures	Acc%	Precision	Recall	F1
Harada et al. [14]	Pressure Matrix + Video camera	Pressure image templates	3	~	~	~	~
Clever et al. [4]	Pressure Matrix	ConvNets	3D Posture	~	~	~	~
Heydarzadeh et al. [9]	Pressure Matrix	GMM + kNN	4	98.10%	~	~	~
Grimm et al. [10]	Pressure Matrix Range Matrix	kNN kNN	4 4	95.50%79.40%	~	~	~
Enokibori et al. [15]	Pressure Matrix	DNN	4	97.10%	~	~	0.970
Matar et al. [16]	Pressure Matrix	FFANN	4	91.00%	0.979	~	~
Yousefi et al. [11]	Pressure Matrix	PCA + kNN	5	97.70%	~	~	~
Hsia et al. [17]	Pressure Matrix	SVM	6	83.50%	~	~	~
Liu et al. [18]	Pressure Matrix	Minimum class residual	6	83.50%	0.831	0.829	0.832
Pouyan et al. [12]	Pressure Matrix	Hamming distance + kNN	8	97.10%	~	~	~
Huang et al. [19]	Pressure Matrix + Video camera	PCA + SVM	9	94.05%	~	~	~
Ren et al. [20]	Kinect v2	Fuzzy + SVM	20	97.10%	~	~	~

Note: All results are based on the weighted average. Some prior work had individual metric scores rather than the weighted average and were therefore dropped for comparison. ConvNets = convolutional networks, GMM = Gaussian mixture model, kNN = k-nearest neighbor, DNN = deep neural network, FFANN = feed-forward artificial neural network, PCA = principal component analysis, SVM = support vector machine.

**Table 2:** Overview of the ten postures that were analyzed with the ConvLSTM model (extracted postures are from [23]).

Index	Posture	Index	Posture
0	Supine	5	supine raised
1	Right	6	supine right raised
2	Left	7	supine left raised
3	Supine wide	8	right fetus
4	Supine straight	9	left fetus

**Table 3:** Performance results of the ConvLSTM.

Posture	Precision	Recall	F1
0	0.9801	0.9979	0.9889
1	0.9929	0.9915	0.9922
2	0.9941	0.9854	0.9898
3	0.9923	0.9848	0.9885
4	0.9683	0.9760	0.9721
5	1.0000	0.9713	0.9854
6	0.9919	0.9880	0.9899
7	0.9917	0.9835	0.9876
8	0.9706	0.9900	0.9802
9	1.0000	0.9827	0.9913
WA	0.9881	0.9880	0.9880

**Table 4:** Fuzzy Inference Output.

HoPB_num	Settings	HoPB_num	Settings
1	0	11	−0.94796
2	0.00013	12	0.58079
3	0.18079	13	0.63512
4	0.63512	14	0.00013
5	−0.91529	15	−0.98468
6	−0.64587	16	−0.70050
7	0.63512	17	0.00013
8	0.70013	18	−0.64587
9	−0.84087	19	0
10	−1.11740	20	0

**Table 5:** Performance results of the ConvLSTM and Fuzzy Inference.

HoPB No.	Decubitus	Ulcer Level	Time	Optimized Setting
1	1	0	0	0
2	1	5	9	0.00013
3	1	3	2	0.18079
4	1	3	5	0.63512
5	1	16	9	−0.91529
6	1	18	12	−0.64587
7	1	3	7	0.63512
8	1	1	10	0.70013
9	1	18	14	−0.84087
10	1	20	13	−1.11740
11	1	5	15	−0.94796
12	1	3	3	0.58079
13	1	3	7	0.63512
14	1	3	8	0.00013
15	1	14	7	−0.98468
16	1	13	7	−0.70050
17	1	3	8	0.00013
18	1	8	8	−0.64587
19	1	0	0	0
20	1	0	0	0

## Data Availability

The data presented in this study are available on request from the corresponding author. The data are not publicly available as permission must first be obtained from the funding institutions to the corresponding author before being shared to the public.
